# Beyond antibiotics: probiotics as a promising ally against *Helicobacter pylori*

**DOI:** 10.3389/fphar.2025.1620870

**Published:** 2025-07-11

**Authors:** Lin Yuan, Chong Yang, Ying Han, Fan Yang, Huabing Tu

**Affiliations:** ^1^ Kweichow Moutai Distillery Co., Ltd., Renhuai, China; ^2^ Kweichow Moutai Group, Guizhou Key Laboratory of Microbial Resources Exploration in Fermentation Industry, Zunyi, China

**Keywords:** *Helicobacter pylori*, antibiotic resistance, probiotic, *lactobacillus*, eradication

## Abstract

*Helicobacter pylori* (*H. pylori*) is considered a key causative agent of gastritis, peptic ulcer, and gastric cancer, affecting more than half of the world’s population. The eradication rate of antibiotic therapy gradually decreases due to the increased risk of resistance. Recent studies have shown that probiotics have good potential in the treatment of *H. pylori* infection. Several studies involving both human and animal models have demonstrated that probiotic interventions can inhibit *H. pylori* growth, attenuate H. pylori-induced gastritis, and enhance the eradication rate of antibiotics while reducing side effects. However, there remains some debate regarding the effective benefits of probiotics. The recently published reviews have not systematically elaborated on the differences in outcomes resulting from the use of probiotics of various types and doses, or the combination of probiotics with medications. They have primarily focused on animal studies, without addressing the heterogeneity of results observed in clinical research and the underlying mechanisms, thus failing to provide more high-quality evidence. This review aims to discuss the mechanisms of *H. pylori* infection in humans, the effects of probiotics in treating *H. pylori* infection, and the pathways and molecular mechanisms by which probiotics inhibit *H. pylori*. Future challenges include identifying effective strains, determining optimal doses and treatment durations, standardizing experimental protocols, considering individual variability, and further elucidating the specific molecular mechanisms and long-term impacts of probiotic therapy in *H. pylori* infection.

## 1 Introduction

**FIGURE 1:**
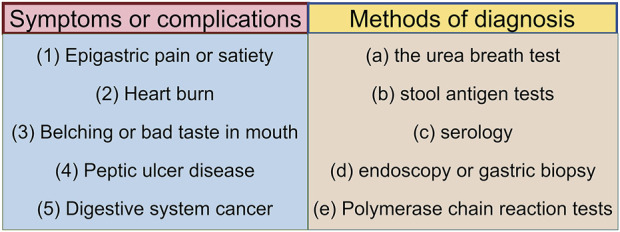
Symptoms after *H. pylori* infection and corresponding diagnostic tools. The relationship between symptoms and diagnostic tools is as follows: symptom (1), corresponds to diagnostic tools (a), (b), and (d); symptom (2), corresponds to diagnostic tools (a) and (d); symptom (3), corresponds to diagnostic tools (a), (b), and (c); symptom (4), corresponds to diagnostic tools (a), (b), and (d); symptom (5), corresponds to diagnostic tools (d) and (e). (By figdraw).

*Helicobacter pylori* (*H. pylori*) is a Gram-negative, microaerophilic spiral bacterium, characterized by its unique morphology and flagella, which enable it to traverse the mucus layer in the acidic gastric environment and adhere to epithelial cells, thereby triggering local inflammatory responses. *Helicobacter pylori* is considered a key pathogenic factor in the development of gastritis, peptic ulcers, mucosa-associated lymphoid tissue lymphoma, and gastric adenocarcinoma (Figure 1) (Li et al., 2024). *Helicobacter pylori* is classified as a group I carcinogen by the World Health Organization. Recently published literature has shown that countries where *H. pylori* infection rates have declined have also seen a decline in the incidence of gastric cancer (Chen Y-C. et al., 2024). Globally, particularly in developing countries and rural areas, the bacterium is transmitted primarily *via* the oral-oral or fecal-oral routes, affecting over half of the population (Duan et al., 2023; Stefano et al., 2018). *Helicobacter pylori* infection typically occurs in childhood, with more than 30% of children being infected (Yuan et al., 2022). Given that children in this early stage of infection are unlikely to develop disease complications, treatment is often considered unnecessary (Saito et al., 2021). Furthermore, *H. pylori* infection may influence pregnancy outcomes (Masaadeh et al., 2023). Clinical diagnostic methods for *H. pylori* infection include the urea breath test, stool antigen test, serum antibody test, and gastric biopsy during endoscopy (Figure 1) (Addissouky et al., 2023).

**FIGURE 2:**
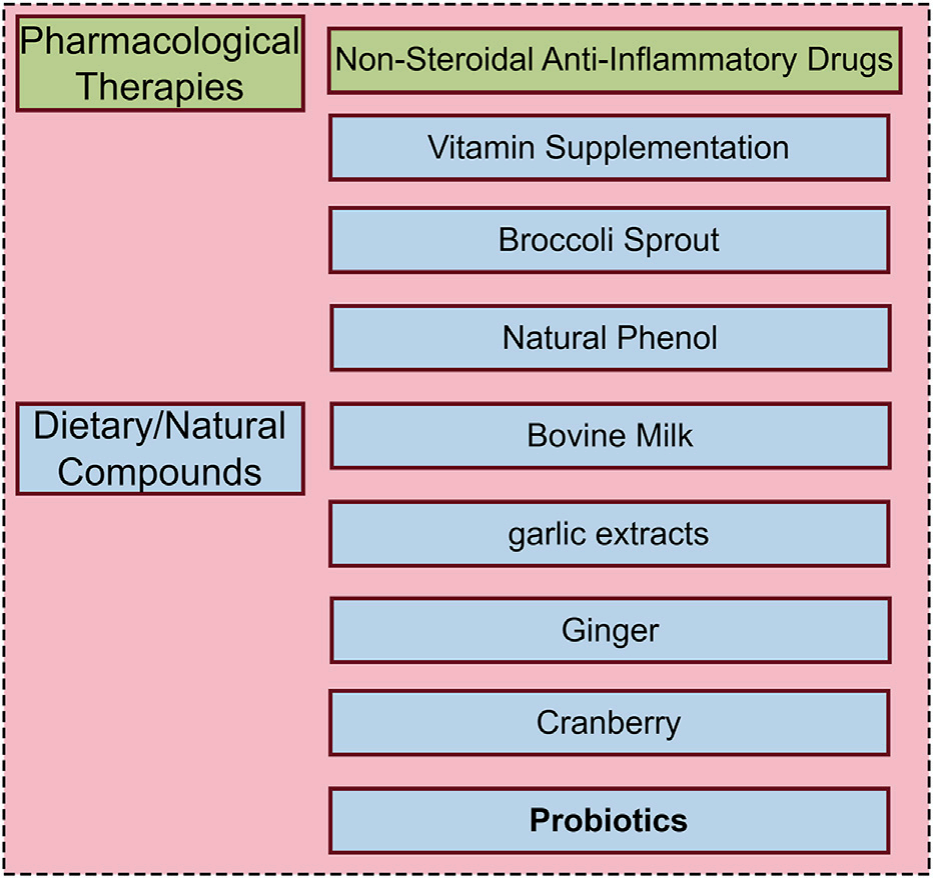
Potential methods for prevention and treatment of *H. pylori* infection.

The standard treatment for *H. pylori* infection involves a triple therapy or quadruple therapy regimen. Triple therapy typically consists of a proton pump inhibitor (PPI) and two antibiotics administered for 7–14 days, while quadruple therapy adds a bismuth agent to enhance eradication rates. Despite the clinical efficacy of these regimens, significant limitations persist. Firstly, antibiotic resistance, particularly to macrolides such as clarithromycin, has been increasing, leading to a higher treatment failure rate (Malfertheiner et al., 2024). By 2050, it is estimated that 10 million people will die due to antimicrobial resistance infection (de Kraker et al., 2016). Secondly, the complexity of the drug regimen and the extended treatment duration contribute to poor patient adherence. Additionally, common adverse effects, such as gastrointestinal discomfort including diarrhea, further reduce compliance (Chen Z. et al., 2024). In the context of complex gastric lesions and infections, a recent review suggested that traction-assisted endoscopic submucosal dissection may improve clinical treatment efficiency (Niu C. et al., 2024); similarly, microbial modulation potentially improving therapeutic outcomes by promoting gastrointestinal function. In response to these challenges, researchers are developing new anti-H. pylori drugs and exploring simpler and more effective treatment regimens, including new antibacterial drugs, natural plant extracts, anti-H. pylori probiotics and some other anti- *H. pylori* foods or drugs (Zimmermann and Curtis, 2017; Takeuchi et al., 2014; El et al., 2023; Maria et al., 2023; Shadvar et al., 2024) (Figure 2). Antibiotic choices for children and pregnant women are limited, and probiotic therapy has emerged as a potential alternative approach.

Probiotics are a group of active microorganisms that are beneficial to host health, mainly including *lactic acid bacteria* and *Bifidobacterium.* Probiotics exert their beneficial effects through various mechanisms, such as competitive inhibition of pathogenic microorganisms, modulation of the gut microbiota, and enhancement of immune functions. Additionally, probiotics reinforce the mucosal barrier, preventing pathogen invasion or producing metabolites (e.g., short-chain fatty acids and antimicrobial peptides) that inhibit the growth of harmful microorganisms. In the treatment of *H. pylori* infection, probiotics demonstrate significant adjunctive roles. Certain probiotic strains can directly inhibit the growth of *H. pylori* or modulate the gastric pH, thereby creating an unfavorable environment for *H. pylori* survival. Furthermore, probiotics can enhance the host’s immune response, facilitating the clearance of *H. pylori*. Consequently, probiotics not only reduce the reliance on antibiotics but also alleviate their side effects, making them a promising alternative strategy for the safe and effective treatment of *H. pylori* infection.

The mechanism of *H. pylori* infection in human body and the role of probiotics in the treatment of *H. pylori* infection have been reviewed in some recent literature (Shadvar et al., 2024; Dash et al., 2024; Huang et al., 2024; Liu et al., 2024), which provides a theoretical basis for the feasibility of probiotics in inhibiting *H. pylori* infection. However, the differences in the results of different types and doses of probiotics, and whether they are combined with drugs have not been systematically described. These reviews focused more on animal experiments and did not sort out the heterogeneous results and related mechanisms produced in clinical research, so as to provide more high-quality evidence and systematic summary. This article aims to systematically review the mechanisms of *H. pylori* infection in humans, the *in vivo* effects of probiotics in the treatment of *H. pylori* infection, and the pathways and molecular mechanisms by which probiotics inhibit *H. pylori*. Additionally, the article will explore future research directions regarding the use of probiotics in the prevention and control of *H. pylori* infection.

## 2 Mechanism and control strategy of *Helicobacter pylori* infection

**FIGURE 3:**
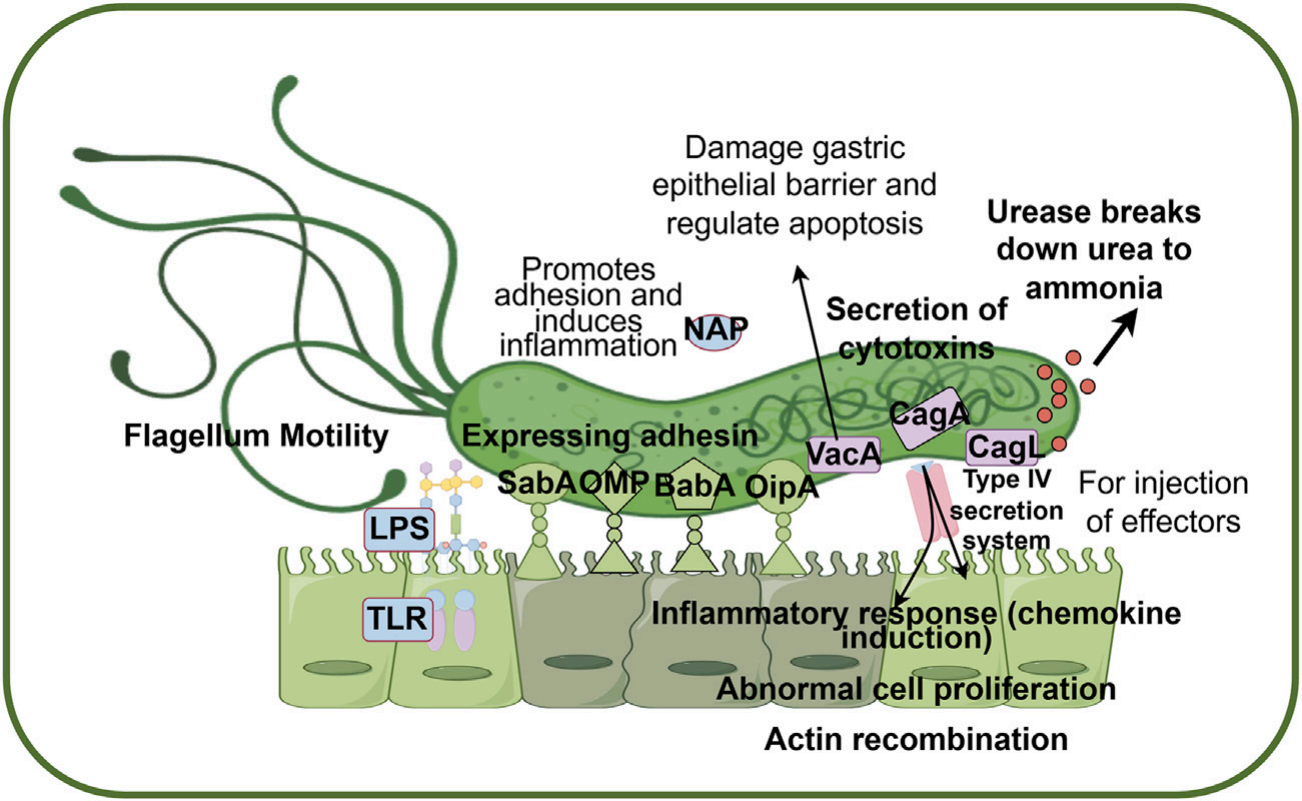
Pathogenic mechanisms of *H. pylori* infection in hosts. Helicobacter pylori passes through the acidic environment of the stomach by producing ammonia and flagellar movement, and produces adhesin to promote adhesion to host epithelial cells. Finally, *H. pylori* secreted a series of toxin proteins to destroy epithelial cells and activate the host inflammatory immune response, resulting in persistent infection. Abbreviations: BabA, blood group antigen-binding adhesion; CagA, cytotoxin-associated gene A; CagL, cytotoxin-associated gene L; NAP, neutrophil-activating protein; OipA, outer inflammatory protein A; SabA, sialic acid binding adhesin; VacA, vacuolating cytotoxin A; LPS, lipolyaccharide; TLR, Toll-like receptor; OMP, Outer membrane protein. Factors coded in green are mainly related to adhesion (BabA, SabA, OipA, OMP), factors coded in pink are mainly related to cytotoxin secretion (CagA, CagL, VacA), and factors coded in blue are mainly related to immune response (LPS, TLR, NAP). (By figdraw).

The pathways of *H. pylori* infection in the host include transmission between genetically related individuals and contact with infected individuals of similar socioeconomic status (Stefano et al., 2018). In recent years, significant progress has been made in understanding the mechanisms of *H. pylori* infection, revealing the complex infection process and immune evasion strategies. The primary process involves *H. pylori* adapting to the acidic gastric environment, releasing adhesion factors that bind to epithelial cells, and subsequently secreting toxin proteins, thereby establishing a persistent infection (Figure 3). Initially, the TlpB receptor on host cells is triggered by chemical signals, promoting a chemotactic response toward the gastric epithelium (Hanyu et al., 2019). *Helicobacter pylori* navigates through the acidic environment *via* its flagella, avoiding direct gastric acid-induced damage, and after traversing the relatively neutral mucus layer, it firmly adheres to gastric epithelial cells (Gu, 2017). *Helicobacter pylori* secretes urease, which hydrolyzes urea to produce ammonia and carbon dioxide, locally neutralizing gastric acid and creating a microenvironment favorable for its survival (Idowu et al., 2022; Elbehiry et al., 2023). Furthermore, *H. pylori* utilizes variably expressed adhesins, such as antigen-binding adhesin (BabA), sialic acid-binding adhesins (SabA), outer inflammatory protein (OipA), and outer membrane proteins (OMP), to facilitate the transition from association with the mucus layer to close adhesion to the epithelial cell layer, thereby preventing the bacterium from being affected by host clearance mechanisms (Sharndama and Mba, 2022). Upon tight binding to gastric epithelial cells, adhesins employ a range of effector proteins, including vacuolating cytotoxin A (VacA), cytotoxin-associated gene A (CagA), and cytotoxin-associated gene L protein (CagL), to manipulate host cell signaling and alter the behavior of gastric epithelial cells, thus promoting long-term colonization (Salama et al., 2013). Among these, the CagA pathogenicity island plays a central coordinating role, injecting CagA into host cells *via* the type IV secretion system, thereby disrupting cellular functions and promoting gastric pathology (Hatakeyama, 2017; Ali and AlHussaini, 2024).

*Helicobacter pylori* establishes persistent infection by modulating the host immune response. Surface antigens of *H. pylori* (e.g., lipopolysaccharides) activate the host’s innate immune system through pattern recognition receptors such as TLR2 and TLR4, while its virulence factors (e.g., VacA and CagA) disrupt epithelial cell integrity, induce apoptosis, and trigger inflammatory responses. The condition for CagA to disrupt *H. pylori* signal transduction pathway is the direct attachment of epithelial cells to integrin-1 (Backert and Tegtmeyer, 2017). After entering the host cell, CagA protein can interact with a variety of signaling molecules, leading to actin reorganization, inflammatory response and abnormal cell proliferation (Shakir et al., 2023). VacA protein is a pore-forming toxin that suddenly kills host cells, interacts with multiple targets, and plays a key role in avoiding immune responses. However, VacA disrupts the integrity of the gastric epithelial barrier by forming channels or pores in the cell membrane, leading to increased permeability, and plays a role in regulating host cell apoptosis according to cell type and environmental conditions (Reyes, 2023; Foegeding et al., 2016). VacA protein also has the function of anti-phagocytosis and producing cytoplasmic vacuoles for *H. pylori* survival (Elbehiry et al., 2023; Baj et al., 2020). VacA toxin contributes to the formation of vacuoles in host cells and affects the structure and function of cells (Foegeding et al., 2016). In addition, NAP conserved protein can promote the adhesion of *H. pylori* to gastric mucosa, and induce the synthesis of IL-8, IL-6, TNF-α and other inflammatory substances to damage gastric mucosa (Zhang et al., 2022). *Helicobacter pylori* colonization, immune escape and causing gastric infection and pathological changes through complex mechanisms. Although the mechanism of infection has been intensively studied, the fine regulation of the interaction between bacteria and the host immune system remains to be revealed.

The detection methods of *H. pylori* infection include fecal antigen detection, urea breath detection and chemical staining of tissue biopsy (Shakir et al., 2023). The treatment guidelines for *H. pylori* in Europe, Canada, the United States, and South Korea recommend the use of quadruple therapy in areas with a resistance rate greater than 15%, whereas Proton pump inhibitor based triple therapy is recommended in areas with a clarithromycin resistance rate of <15%, although this is uncommon (Jung et al., 2021; Deane et al., 2024). The use of quadruple therapy is not allowed in Japan because there is insufficient evidence to prove that it is superior to triple therapy for Japanese patients (Cho and Jin, 2022). Fluoroquinolone-containing drugs are commonly used as second-line treatment for *H. pylori* infection, and a European study involving 5,055 patients receiving second-line treatment showed that 14-day levofloxacin-bismuth treatment was one of the four most effective regimens (Nyssen et al., 2022a). However, due to drug resistance and side effects, the clinical application of fluoroquinolones is limited. Rifabutin therapy is often used as second-,third -, or fourth-line therapy, but it carries the risk of bone marrow suppression and resistance to *Mycobacterium* (Deane et al., 2024; Gisbert, 2020). A European retrospective analysis of the efficacy of Rifabutin triple therapy for the treatment of *H. pylori* infection in 500 patients found eradication rates of 66%–80% (Nyssen et al., 2022b). Antibiotic resistance is one of the main reasons for the decline of *H. pylori* eradication rate. Common resistance mechanisms include some mutations affecting genetically modified drug targets, membrane permeability, efflux pump systems, and biofilm development (Hu et al., 2016; Srisuphanunt et al., 2023). While the gene encoding virulence factor VacA mentioned above was found to be associated with metronidazole resistance, and CagA was associated with levofloxacin resistance (Wang et al., 2019). In the face of increasing antibiotic resistance, it is necessary to revolutionize the eradication treatment of *H. pylori* infection. It is the future research direction to find personalized, effective and sustainable methods to face this global health challenge (Yamaoka, 2024).

## 3 Inhibitory effect of probiotics on *Helicobacter pylori*

*Helicobacter pylori* infection can affect symptoms such as intestinal discomfort, of which the disorder of intestinal microbiota is considered to be one of the main factors. In particular, some pathogens such as *Haemophilus* and *Streptococcus* increased while *Faecalibacterium*, *Lactobacillus*, and *Akkermansia* were reported to decrease significantly (Li et al., 2022; Li et al., 2023). Therefore, probiotic supplementation helps to inhibit *H. pylori* and is also expected to help regulate intestinal microbiota, which is beneficial to relieve gastrointestinal discomfort and help restore gastrointestinal health. Strains represented by *Limosilactobacillus reuteri* DSM 17648 have provided substantial scientific evidence. From the perspective of clinical evidence, *Limosilactobacillus reuteri* DSM 17648 has been proved to enhance the effect of triple and quadruple therapy in the treatment of *H. pylori* infection and reduce side effects (Liang et al., 2022).

### 3.1 *In vivo* and *in vitro* experiment

Sun et al. isolated four strains of lactic acid bacteria (*Lactobacillus sake*, *Lactobacillus plantarum*, *Lactobacillus rhamnosus*, and *Lactobacillus brevis*) from fermented foods in Northeast China, all of which were found to inhibit the growth of *H. pylori* to varying degrees (Sun et al., 2018). *Lactobacillus* paracasei HP7 demonstrated inhibitory effects against *H. pylori* both *in vitro* and *in vivo*. When combined with extracts of Perilla frutescens and Glycyrrhiza uralensis, it alleviated gastric inflammation and mucosal lesions in H. pylori-infected mice (Lee and Kim, 2020). A study on *lactic acid bacteria* isolated from fermented cocoa juice showed that 65.52% of the strains exhibited inhibitory effects against *H. pylori*, with some exerting their action through bacteriocins or organic acids (Kouitcheu Mabeku et al., 2020). *Lactobacillus casei* T1 and its supernatant exhibited potent inhibition of *H. pylori* growth, preventing inflammation and dysbiosis caused by *H. pylori* infection (Wu et al., 2021). The addition of probiotic *Lactobacillus salivarius* LN12 to amoxicillin and clarithromycin enhanced the therapeutic efficacy of the triple therapy, especially against *H. pylori* biofilm (Jin and Yang, 2021). The probiotic *Lactiplantibacillus pentosus* SLC13 has been shown to inhibit *H. pylori* growth, and its extracellular polysaccharides significantly reduced the expression of interleukin 8 (IL-8) induced by *H. pylori* infection, demonstrating its potential as an alternative treatment for *H. pylori* infection and inflammation reduction (Thuy et al., 2022). *Lactiplantibacillus plantarum* ZJ316 exhibited inhibitory effects on *H. pylori* both *in vitro* and *in vivo*, with mechanisms including the prevention of *H. pylori* colonization, downregulation of virulence genes, and reduction of IL-8 production (Wu et al., 2023). Recently, Chen et al. isolated five novel gastric-derived *Weizmannia coagulans* strains from healthy gastric mucosa, among which BCF-01 showed the strongest adhesion and inhibition of *H. pylori* growth. It effectively restored gastric microbiota, improved H. pylori-mediated mucosal barrier disruption, and alleviated inflammation by inhibiting the macrophage TLR4-NFκB-pyroptosis signaling pathway (Chen Z. et al., 2024). Xu et al. isolated *Lactobacillus paragasseri* strain LPG-9 from gastric mucosa, which demonstrated good inhibitory effects on *H. pylori* both *in vitro* and *in vivo*. This strain repaired the mucosal barrier by upregulating the expression of mucosal barrier proteins occludin and ZO-1, alleviating gastritis (Xu et al., 2023). Samy M et al. conducted a screening of different probiotic strains antagonistic to *H. pylori* and found that *Bifidobacterium lactis* and *Lactobacillus acidophilus* exhibited the highest inhibitory effects (Abdelhamid et al., 2023). In conclusion, probiotics show good inhibitory effect on *H. pylori in vitro* and *in vivo* through a variety of mechanisms, especially *Lactobacillus* species. Although probiotics have shown promising prospects in the inhibition of *H. pylori*, more scientific evidences and clinical cohorts are needed to verify their widespread application.

### 3.2 Related clinical study

The meta-analysis conducted by Yang et al. demonstrated that supplementation with probiotics as an adjunctive therapy significantly improved eradication rates (RR 1.10, 95% CI 1.06–1.14) and reduced the overall risk of side effects (RR 0.54, 95% CI 0.42–0.70) compared to standard therapy. Among the probiotics, *Bifidobacterium spp*. exhibited the highest eradication potential, though further high-quality studies are needed (Yang et al., 2024). Another high-quality meta-analysis, based on 40 studies involving 8,924 patients, found an eradication rate of 81.5% following probiotic treatment with various regimens, compared to only 71.6% in the control group (p < 0.001, *I* ^2^ = 52.1%). Probiotic use before and throughout the eradication treatment, especially when lasting more than 2 weeks, showed superior outcomes, with the best results observed when probiotics were combined with bismuth quadruple therapy (Shi et al., 2019). *Lactobacillus acidophilus*, *Lactobacillus plantarum*, and *Lactobacillus rhamnosus* were found to improve gastritis induced by *H. pylori* infection to varying degrees *L. acidophilus* (Asgari et al., 2020). Research by Saracino et al. (2020) showed that *Lactobacillus casei*, *Lactobacillus paracasei*, *Lactobacillus acidophilus*, *B. lactis*, and *Streptococcus thermophilus* exhibited antimicrobial and bactericidal activity against *H. pylori*. However, a meta-analysis of 11 studies involving 403 patients revealed that the average weighted eradication rate for probiotic treatment (including *Lactobacilli* and *Saccharomyces boulardii*) was only 14% (95% CI: 2%–25%, p = 0.02) (Losurdo et al., 2018). Overall, as shown in Table 1 (*In vivo* study), most clinical studies have shown that probiotics increase the eradication rate of *H. pylori* with antibiotics and attenuate treatment-related side effects. However, some studies suggested that the use of the same kind of probiotics did not increase the eradication rate or reduce the side effects. For example, among the studies using *Lactobacillus reuteri* (n = 5), some studies showed that eradication rate could be increased to 65.22%, while others showed no significant effect (n = 2). The reasons for these invalid or contradictory findings may be related to the heterogeneity of studies and the different doses of strains used.

**TABLE 1:** Clinical trial of probiotics for treatment of *H. pylori* infection.

Year	Species of probiotics	Dose/CFU	Medicine	Results	References
2016	*Lactobacillus* Rosell-52, *Lactobacillus* Rosell-11, *Bifidobacterium* Rosell-1755 and *Saccharomyces boulardii*	5 billion live probiotic/capsule	Probiotics plus standard clarithromycin triple therapy	Eradication (probiotics VS control = 93.3% VS 81.8%),incidence of adverse effects (probiotics VS control = 17.7% VS 28.6%)	Grgov et al. (2016)
2017	*Lactobacillus* and *Bifidobacterium*	with total viable count of 15 × 10^8^ CFU/capsule.	double strain probiotic combination with standard triple therapy	Eradication (probiotics VS control = 78.4% VS 64.8%) has increased, incidence of adverse events was significantly reduced	Haghdoost et al. (2017)
2018	*Lactobacillus plantarum* and *Pediococcus acidilactici*	1 × 10^9^ CFU/strain	probiotic combination with triple or nonbismuth quadruple	Neither reduced the side effects nor improved the eradication rate	McNicholl et al. (2018)
2019	*Lactobacillus reuteri* DSMZ17648	NA	esomeprasole and the association between probiotic, deglycyrrhizinated liquorice extract and calcium carbonate	The eradication efficiency was 54.3%. The rate of side effects was significantly lower than that of antibiotic group (2.9% vs. 17.1%).	Mihai et al. (2019)
2019	*Lactobacillus reuteri*	NA	probiotic plus pantoprazole	Eradication (probiotics VS control = 65.22% VS 73.91%)	Muresan et al. (2019)
2020	*Bacillus clausii*	2 × 10^9^ CFU	probiotic combination with triple therapy	reduced the incidence of, and the number of days with, diarrhea in patients receiving *H. pylori* eradication therapy	Dorothea and Greifenberg (2020)
2020	*Saccharomyces boulardii*	3 × 10^10^ CFU/g, 3 capsules/day	Probiotics plus standard clarithromycin triple therapy	neither increased the eradication rate nor reduced the occurrence of adverse events	Chang et al. (2020)
2021	*Lactobacillus acidophilus* and *Lactobacillus rhamnosus*	3 × 10^9^ CFU/sachet, 2 sachets/day	Probiotics	the bacterial load of *H. pylori* reduced	Chen et al. (2021b)
2021	*Lactobacillus reuteri* DSMZ17648	1 × 10^10^ dead cells/packet, 4 packets/day	non- viable probiotic combination with triple therapy	did not improve the eradication rate of *H. pylori*; reduced the frequencies of abdominal distention and diarrhea	Yang et al. (2021)
2022	*Lactobacillus Acidophilus, Lactiplantibacillus plantarum, Bifidobacterium lactis*, and *Saccharomyces boulardii*	1.75 × 10^9^, 5 × 10^8^, 1.75 × 10^9^, and 1.5 × 10^9^; twice daily	probiotic combination with non-bismuth quadruple therapy	increases the eradication rate of *H. pylori* and decreases side effects	Viazis et al. (2022)
2023	*Enterococcus faecium* and *Bacillus subtilis*	4.5 × 10^8^ and 5.0 × 10^7^	probiotic	Supplementation with probiotics after triple therapy did not increase eradication rates or reduce recurrence rates	Lim et al. (2023)
2023	*Lactobacillus reuteri* DSM 17648	4.0 × 10^9^ CFU	Probiotics combined with triple therapy	The eradication rate of *H. pylori* increased significantly and the side effects decreased	Ismail et al. (2023)
2023	*Lactobacillus ruteri*	NA	Probiotics combined with quadruple therapy	did not significantly improve the eradication, but reduced the frequency of drug-associated side effects	Mohtasham et al. (2023)
2024	*Saccharomyces boulardii* CNCMI-745	NA	Probiotics combined with triple therapy	There was no significant increase in eradication rates and significant reduction in diarrhea	Sjomina et al. (2024)
2024	*Lacticaseibacillus rhamnosus* LRa05	1 × 10^10^ CFU	quadruple therapy combined probiotic	Reduce side effects of quadruple therapy and improve the eradication rate of *H. pylori*	Niu et al. (2024b)

In conclusion, current clinical research on the antagonism of *H. pylori* primarily focuses on certain strains of *Lactobacillus*, *Bifidobacterium*, and *Saccharomyces*. These probiotics have been shown to reduce *H. pylori* infection rates and attenuate gastrointestinal symptoms, while also enhancing the efficacy of antibiotic treatments for *H. pylori* infection (Baryshnikova et al., 2023). As one of the most widely used probiotics, *Lactobacillus* has been demonstrated to decrease *H. pylori* colonization and attenuate gastrointestinal discomfort. When combined with antibiotics, it can effectively improve the eradication rate of *H. pylori* and reduce adverse reactions during treatment. Specifically, *Lactobacillus reuteri* produces potent antimicrobial substances and secretes mucin to strengthen the mucosal barrier, showing promising inhibitory effects against *H. pylori* (Dargenio et al., 2022; Dargenio et al., 2021). The ability of probiotics to mitigate the side effects of antibiotics may be related to their regulation of the gut microbiota. Li et al.'s analysis revealed that the enrichment of *H. pylori* in the stomach affects the composition of the gastrointestinal microbiota (Li et al., 2023). Similarly, Bai et al.'s findings suggest that probiotics contribute to the balance of the intestinal microbiota, attenuating gastrointestinal discomfort, although more data are needed to determine whether they can increase eradication rates (Bai et al., 2023). However, it is important to point out that the above conclusions are all based on previously published articles, and there may be heterogeneity among different studies. In particular, the heterogeneity of studies, publication bias or the quality of included studies should be fully considered when referring to meta-analysis (Niu C. et al., 2024).

## 4 Mechanism of probiotics inhibiting *Helicobacter pylori*

### 4.1 Competitive exclusion

*Helicobacter pylori* colonizes the gastric mucosa by utilizing adhesion factors, such as outer membrane proteins (OMP). While most lactic acid bacteria colonize the human intestine, a few species are capable of colonizing the stomach (Ji and Yang, 2020). Probiotics can compete with *H. pylori* for adhesion sites on gastric epithelial cells, thereby inhibiting its colonization. For example, *S. boulardii* can prevent the binding of *H. pylori* to host cells (mainly through the modification of *H. pylori* binding sites on duodenal cells by ceramidase (Czerucka and Rampal, 2019). *Lactobacillus rhamnosus* ATCC 7469, L. acidophilus ATCC 4356, and L. reuteri ATCC 23272 were found to inhibit the adhesion of *H. pylori* to gastric epithelial cells (Rezaee et al., 2019). In addition, probiotics can modify the expression of epithelial junction proteins and mucins, and release active substances to protect mucosal barrier damage and prevent *H. pylori* colonization (Qureshi et al., 2019).

### 4.2 Regulation of gastric acid environment

The urease secreted by *H. pylori* breaks down urea into ammonia, locally neutralizing the acidic gastric environment to facilitate its survival. Probiotics, on the other hand, counteract this by producing lactic acid or inhibiting urease activity, thereby preventing an increase in gastric pH and weakening *H. pylori*’s survival mechanisms within the stomach. *Lactobacillus* plantarum ZJ316 can suppress the expression of the urease gene in *H. pylori*, thereby preventing its colonization (Wu et al., 2023). *Lactobacillus acidophilus* ATCC 4356, L. reuteri ATCC 23272 and *L. fermentum* ATCC 9338 could reduce the urease activity of eight clinical *H. pylori* strains (Rezaee et al., 2019). *Lactobacillus* rhamnosus GMNL-74 and *Lactobacillus* acidophilusGMNL-185 can inhibit the adhesion of *H. pylori* to gastric epithelium and reduce inflammation caused by infection (Chen et al., 2019). In addition, *H. pylori* can further weaken gastric mucosal barrier by interfering with intragastric acid-base balance and changing gastric acid secretion, and increase the risk of peptic ulcer and other pathogen infection.

### 4.3 Production of antimicrobial substances

Probiotics can produce various antimicrobial substances through biological metabolism. Lactobacilli, for example, metabolize carbohydrates to generate short-chain fatty acids such as acetic acid, propionic acid, butyric acid, and other organic acids. They can also produce bacteriocins and hydrogen peroxide, all of which exhibit significant antimicrobial properties (Kim et al., 2003; Homan and Orel, 2015). The organic acids metabolized by lactobacilli not only lower the gastric pH but also inhibit the activity of urease, thereby hindering the growth of *H. pylori* (Rezaee et al., 2019). Research by Rezaee et al., 2019 confirmed that *Lactobacillus reuteri* ATCC 23272 can inhibit *H. pylori* by producing antimicrobial acids. A recent study demonstrated that *Weizmannia coagulans* BC99 improved inflammation and oxidative stress after *H. pylori* infection by modulating gut microbiota-derived metabolites such as valeric acid (Zhai et al., 2024). Hydrogen peroxide produced by probiotics can cause oxidative damage of *H. pylori* cells by inducing the production of peroxide ions and interfering with *H. pylori* activity (Ji and Yang, 2020; Bai et al., 2022). The antioxidant system within *H. pylori* itself can produce enzymes such as superoxide dismutase to counteract host immune responses (Bereswill et al., 2000). Bacteriocins can disrupt the cell wall and membrane structures of *H. pylori*. Hu et al. (2021) reported that extracellular polysaccharides produced by *Lactobacillus plajomi* PW-7 effectively inhibit the growth of *H. pylori* and compromise its cell membrane integrity.

*Helicobacter pylori* incubation with supernatant metabolites from L. gasseri resulted in downregulation of acid resistance related gene arsS and flagella regulatory gene flgR, thereby reducing *H. pylori* activity. In addition, *H. pylori* iron absorption regulatory genes were downregulated. Results in a significant increase in sensitivity to antimicrobial peptide LL-37 (Zuo et al., 2022). In conclusion, probiotics can produce a variety of anti-H. pylori antibiotics, but which substances play the main role and whether multiple substances have synergistic effects need further identification and experimental verification. With the vigorous development of synthetic biology, future studies can try to use cloning strategies such as red/ET-mediated homologous recombination and transformation-related recombination to synthesize probiotic metabolites with antagonistic *H. pylori* (Alam et al., 2021).

### 4.4 Immunoregulatory effects

The persistent inflammatory response following *H. pylori* infection may induce inflammatory diseases (de Brito et al., 2019). The regulation of the host immune system by probiotics is conducive to reducing the immune escape of *H. pylori*. Probiotics enhance the host resistance to *H. pylori* chronic infection by regulating dendritic cells to induce B cells to produce Immunoglobulin A (IgA) (Dash et al., 2024). *Lactobacillus gasseri* Kx110A1, isolated from the human stomach, inhibits the expression of tumor necrosis factor-α (TNF-α) converting enzyme on host macrophages, consequently reducing the release of TNF-α and interleukin-6 (IL-6) (Gebremariam et al., 2019). IL-8 induces the migration of neutrophils and monocytes to the mucosa. *Lactobacillus plantarum* ZJ316 protects the host from inflammatory injury by inhibiting immune cell infiltration and IL-8 production during *H. pylori* infection (Wu et al., 2023). Research evidence suggests that probiotics may also enhance the expression of anti-inflammatory cytokine interleukin-10 (IL-10) (Zhao et al., 2018). A study by Park et al. (2020) demonstrated that, compared to the *H. pylori* infection group, the group of mice treated with *Lactobacillus plantarum* APSulloc 331,261 exhibited a significant downregulation of inflammatory cytokines such as TNF-α, interleukin-1β (IL-1β), and interleukin-4 (IL-4). *Lactobacillus plantarum* ZJ316 was found to significantly reduce the levels of interferon-γ and IL-6, increase the level of IL-10, and repair mucosal damage, thereby reducing *H. pylori* abundance and attenuating gastric inflammation caused by *H. pylori* infection (Zhou et al., 2021). *Lactobacillus acidophilus* NCFM and *Lactiplantibacillus plantarum* Lp-115 improved *H. pylori* eradication rate and attenuated gastric inflammation. This result is associated with an immunomodulatory process (reduced expression of cytokines such as IL-8 and TNF-α) (Shen et al., 2023). *Lactobacillus fermentum* UCO-979C enhances resistance to *H. pylori* infection by modulating the gastric innate immune response, significantly reducing the levels of TNF-α, IL-8, and Monocyte Chemotactic Protein 1 (MCP-1) in the gastric mucosa of infected mice, while increasing the expression of Interferon-gamma (IFN-γ) and IL-10 (Garcia-Castillo et al., 2020). *Lactobacillus gasseri* ATCC 33323 inhibits the secretion of IL-8 in human gastric adenocarcinoma cells infected with *H. pylori*, and significantly reduces the mRNA expression of genes such as Bcl-2, β-catenin, integrin α5, and integrin β1 (Yarmohammadi et al., 2021). *Lactobacillus rhamnosus* JB3 suppresses IL-8 secretion, as well as the mRNA levels of vacA, sabA, and fucT, and the expression of Lewis (Le)x antigens and Toll-like receptor 4 (TLR4) in H. pylori-infected AGS cells (Do et al., 2021). Both live and pasteurized *Lactobacillus crispatus* strain RIGLD-1 regulate H. pylori-induced inflammation by downregulating the mRNA expression of IL-1β, IL-6, IL-8, and TNF-α, and upregulating the expression of IL-10 and Transforming Growth Factor Beta (TGF-β) cytokines in AGS cells (Fakharian et al., 2023). Lin et al. (2020) investigated the effects of multiple *Lactobacillus* species on the immune response and metabolic balance of *H. pylori* infected mice. The results showed that the intervention of multiple *Lactobacillus* species could restore the levels of alanine, arginine, aspartic acid, glycine and tryptophan in serum, and increase the contents of butyric acid, valeric acid, palmitic acid, palmitic acid, stearic acid and oleic acid. These are important indicators related to immunity and metabolism (Lin et al., 2020).

In conclusion, probiotics promote the activation of immune cells in the host gastric mucosa, increase the secretion of cytokines such as IL-10 and IgA, and enhance the host immune defense function. In addition, probiotics such as *Lactobacillus* and *Bifidobacterium* can also help to clear *H. pylori* by regulating innate immunity, enhancing immune response and strengthening mucosal barrier.

### 4.5 Regulation of gastric microecology

**FIGURE 4:**
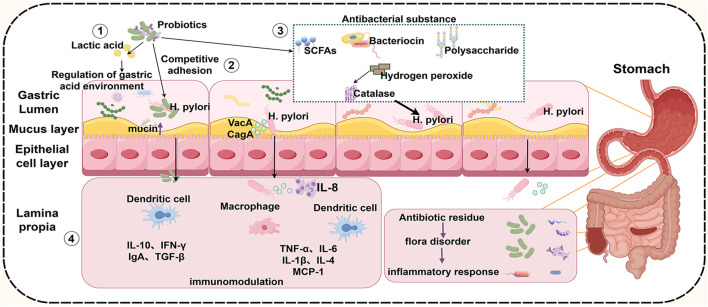
Mechanisms by which probiotics inhibit *H. pylori*. Probiotics improve the gastrointestinal microecological environment by changing the gastric pH value ①, competitive adhesion with *H. pylori* ②, secreting antibacterial substances③, increasing the secretion of anti-inflammatory factors and reducing the secretion of pro-inflammatory factors ④, thereby attenuating *H. pylori* infection and reducing related complications. SCFAs, short chain fatty acids; IL-10, interleukin-10; IFN-γ, Interferon-gamma; IgA, Immunoglobulin A; TGF-β, Transforming Growth Factor Beta; TNF-α, tumor necrosis factor-α; IL-6, interleukin-6; IL-1β, interleukin-1β; IL-4, interleukin-4; MCP-1, Monocyte Chemotactic Protein-1; VacA, vacuolated cytotoxin), CagA, bacterial toxin-associated gene A. (By figdraw).

The analysis of data from GEO revealed significant differences in the microbiota between healthy individuals and those infected with *H. pylori*. Furthermore, 11 bacterial populations that were significantly negatively correlated with *H. pylori* infection and notably enriched in the HP- group were identified, the majority of which were probiotic species, including *Lactobacillus* and *Enterococcus* (Chen Z. et al., 2021). Compared with gut microbiota, gastric microbiota is characterized by low density, poor specificity, and large fluctuations. The number of bacteria in the gastric microecosystem of healthy adults varies significantly, which may be affected by many factors such as region, culture and dietary habits (Xu et al., 2022; Stewart et al., 2020). He et al. found that the combination of *Lactobacillus salivarius* and *Lactobacillus rhamnosus* could improve the gastric and intestinal microecology in the *H. pylori* infected group. n particular, H. pylor-induced reduction of anti-inflammatory bacteria Faecalibaculum in the intestine was restored, and inflammatory infiltration and the incidence of precancerous lesions were reduced (He et al., 2022). The supplementation of probiotics can also regulate the structure of gastric flora, and the recovery of gastric microecology is considered to be related to the eradication of *H. pylori* (Nabavi-Rad et al., 2022). The quadruple therapy of antibiotics in the treatment of *H. pylori* infection can aggravate the gastrointestinal microecological disorder. While probiotics restore the balance of intestinal flora through different mechanisms, improve the eradication rate, and reduce the occurrence of adverse reactions (Xu et al., 2022). In conclusion, probiotics play an important role in the occurrence and development of gastrointestinal diseases by antagonizing *H. pylori* colonization by regulating gastric pH, secreting antibacterial substances, stimulating immune responses and regulating gastrointestinal flora (Figure 4).

## 5 Conclusions and future perspectives

This review systematically summarizes the mechanisms of *H. pylori* (*H. pylori*) infection and its prevention and treatment strategies, with a particular focus on the latest research advancements regarding the role of probiotics in the management of *H. pylori* infection. Emphasis is placed on the mechanisms by which probiotics inhibit *H. pylori* and the directions for future research. Numerous studies have indicated that probiotic supplementation is beneficial in preventing and treating *H. pylori* infection. However, the effectiveness of probiotic therapies in eradicating *H. pylori* has been inconsistent, with low eradication rates, which may be attributed to the unique gastric environment that hampers the colonization of probiotics derived from the intestinal tract. Therefore, it is crucial to identify probiotics that can withstand the acidic gastric environment and effectively eradicate *H. pylori*. Autologous probiotics are considered a promising new approach in microbial therapy, demonstrating good efficacy in inhibiting *H. pylori*, but further validation through large, multicenter, randomized controlled trials is still required (Baryshnikova et al., 2023). Although probiotics have been shown to be beneficial in selected settings (e.g., antibiotic-associated diarrhea and certain types of inflammatory bowel disease), their routine use or as adjunctive therapy for *H. pylori* in healthy individuals is not supported by strong clinical evidence. Given the uncertainties regarding the effectiveness of probiotics in treating *H. pylori* infection, some meta-analyses have even reached contradictory conclusions. The Maastricht VI/Florence Consensus Report (2021) does not recommend routine probiotic supplementation due to inconsistent clinical evidence regarding efficacy (Malfertheiner et al., 2022). The 2020 guidelines of the American Gastroenterological Association also state that probiotics are not recommended for most gastrointestinal diseases (Su et al., 2020).

Probiotic treatment may also lead to adverse effects, such as exacerbated constipation and bloating, and its safety profile requires further investigation. Moreover, although *Enterococcus* spp. is widely used in food and feed, its ability to harbor virulence factor and inherent antimicrobial resistance raises significant safety concerns. Of note, *Enterococcus* are excluded from the US FDA GRAS list and the EU QPS list (Franz et al., 2011). Therefore, strain identification and description, production process and quality control, clinical trials and efficacy verification, safety assessment, clear labeling and instructions, and continuous monitoring and feedback mechanisms need to be further improved in the regulatory aspect of the safety of probiotics use.

Nevertheless, for patients who experience severe side effects from antibiotic therapy or have limited antibiotic options, as well as those with susceptible gastrointestinal microbiota, probiotic therapy offers a promising alternative. Future research should differentiate between symptomatic and asymptomatic infected individuals, select specific probiotics or their fermented products, utilize widely studied strains, standardize experimental protocols (including dosage, duration, and clinical endpoints), and assess the long-term effects of probiotic interventions.
